# Complete Genome Sequence of Streptococcus mitis Strain Nm-65, Isolated from a Patient with Kawasaki Disease

**DOI:** 10.1128/MRA.01239-20

**Published:** 2021-01-07

**Authors:** Atsushi Tabata, Hisashi Ohkuni, Yasuhiko Itoh, Yoshitaka Fukunaga, Toshifumi Tomoyasu, Hideaki Nagamune

**Affiliations:** a Department of Bioscience and Bioindustry, Graduate School of Technology, Industrial and Social Sciences, Tokushima University, Tokushima, Tokushima, Japan; b Health Science Research Institute East Japan, Kounosu, Saitama, Japan; c Department of Pediatrics, Nippon Medical School, Tokyo, Japan; University of Rochester School of Medicine and Dentistry

## Abstract

Streptococcus mitis Nm-65 is a human commensal streptococcal strain of the mitis group that was isolated from the tooth surface of a patient with Kawasaki disease. The complete genome sequence of Nm-65 was obtained by means of hybrid assembly, using two next-generation sequencing data sets. The final assembly size was 2,085,837 bp, with 2,039 coding sequences.

## ANNOUNCEMENT

Streptococcus mitis inhabits the human oral cavity and is considered an opportunistic pathogen of increasing clinical importance (1–5). Strain Nm-65 was isolated from a patient with Kawasaki disease at Nippon Medical School Hospital (Tokyo, Japan) in 1988 with the patient’s consent and was used according to ethical guidelines provided by the Japanese Society for Bacteriology. Identification of Nm-65 was conducted as described previously (6). Nm-65 was then cultured overnight in brain heart infusion broth (Becton, Dickinson, Franklin Lakes, NJ, USA) at 37°C (in 5% CO_2_, 75% N_2_, and 20% O_2_), following inoculation of glycerol stock prepared from the originally passaged single colony. Genomic DNA was prepared as described previously (7), and both a short-read sequencer (454 GS FLX; Roche, Basel, Switzerland) and a long-read sequencer (MinION; Oxford Nanopore Technologies, Oxford, UK) were used. For 454 GS FLX sequencing (outsourced to Hokkaido System Science Co., Ltd., Hokkaido, Japan), the library was constructed using the GS FLX Titanium general library preparation kit (Roche). Sequencing was then conducted using the GS FLX Titanium SV emPCR kit (Lib-L) (Roche) and the GS FLX Titanium XLR70 sequencing kit (Roche) (run parameters: XLR70, 200 cycles). The base-calling software was GS Run Processor v2.3 (Roche). For MinION sequencing, the library was constructed using a rapid sequencing kit (Oxford Nanopore Technologies). Sequencing was then conducted using a MinION system with an R9 MinION flow cell (Oxford Nanopore Technologies). The base-calling software was MinKNOW v3.3.2 (Oxford Nanopore Technologies), and sequences were assembled using NanoTools v1.0 software (https://github.com/WorldFusion/nanotools/blob/master/README.md) (World Fusion Co., Ltd., Tokyo, Japan). Using the acquired sequences (122,996 reads [representing 43,667,782 bp] provided by short-read sequencing and 119,632 reads [with an *N*_50_ value of 12.82 kb] provided by long-read sequencing), a hybrid assembly was generated by the Taniguchi Dental Clinic/Oral Microbiome Center (Kagawa, Japan). Low-quality reads (with a score of ≤Q15 for short-read sequencing and ≤Q10 for long-read sequencing), short reads (≤10 bp for short-read sequencing and ≤1,000 bp for long-read sequencing), and adaptor sequences were removed using fastp v0.20.0 software (8) for short-read sequences and NanoFilt v.2.7.1 software (9) for long-read sequences. The remaining high-quality reads (43,259,318 bp [∼20.7× coverage] derived from short-read sequencing and 899,765,713 bp [∼431.4 × coverage] derived from long-read sequencing) were then assembled using Unicycler v0.4.8 software (10) and were visualized using Bandage v0.8.1 software (11) to confirm a closed circular sequence. The assembled sequence was polished using Pilon v1.23 software (12). For the analyses in this study, all software was operated using default settings and parameters unless otherwise specified.

The resultant complete Nm-65 genome sequence is 2,085,837 bp long and exhibits a GC content of 40.0%, with 2,039 coding sequences (coding proportion, 87.3%) as predicted by DFAST (https://dfast.nig.ac.jp), prophage regions as predicted by PHASTER (https://phaster.ca) (Table 1), and a single CRISPR-Cas system (SMNM65_07910 [GenBank accession number BCJ10359.1] to SMNM65_08010 [BCJ10369.1]) as predicted by CRISPRCasFinder (https://crisprcas.i2bc.paris-saclay.fr/CrisprCasFinder/Index). The genes encoding cholesterol-dependent cytolysins (CDCs), S. mitis-derived human platelet aggregation factor (13, 14), and mitilysin (15, 16) are distinct from the prophage regions and the CRISPR-Cas system. Since the S. mitis type strain does not possess CDC genes, elucidating the Nm-65 mechanisms for acquiring genes encoding such virulence factors may improve the understanding of the opportunistic pathogenicity exhibited by certain S. mitis strains. Such information may be relevant to cryptogenic infections, including those in the context of Kawasaki disease.

**TABLE 1:** Predicted prophage regions present in the S. mitis Nm-65 genome

Genome nucleotide position	Completeness	Score^*a*^	No. of open reading frames	GC content (%)	Most similar phage (GenBank accession no.)
22596–62556	Questionable	81	58	40.4	*Streptococcus* phage PH10 (NC_012756.1)
535758–562399	Incomplete	20	23	40.5	*Streptococcus* phage Dp-1 (NC_015274.1)
914783–923305	Incomplete	40	8	41.1	*Bacillus* phage AR9 (NC_031039.1)

a A score of 70 to 90 indicates a questionable result, and a score of <70 indicates an incomplete result.

### Data availability.

This complete genome sequence of S. mitis strain Nm-65 has been deposited in DDBJ/ENA/GenBank under accession number AP023349. The associated BioProject and BioSample numbers are PRJDB10372 and SAMD00239187, respectively. Additionally, the SRA accession numbers are DRR243499 and DRR243500.
